# Prevalence of *BRCA1* and *BRCA2* Mutations Among High-Risk Saudi Patients With Breast Cancer 

**DOI:** 10.1200/JGO.18.00066

**Published:** 2018-09-10

**Authors:** Omalkhair Abulkhair, Mohammed Al Balwi, Ola Makram, Lamia Alsubaie, Medhat Faris, Hussam Shehata, Ahmed Hashim, Banu Arun, Ahmed Saadeddin, Ezzeldin Ibrahim

**Affiliations:** **Omalkhair Abulkhair**, Specialized Medical Center; **Mohammed Al Balwi**, **Ola Makram**, **Lamia Alsubaie**, **Hussam Shehata**, **Ahmed Hashim**, and **Ahmed Saadeddin**, King Abdulaziz Medical City, Ministry of National Guard–Health Affairs; **Mohammed Al Balwi**, King Abdullah International Medical Research Center, Ministry of National Guard–Health Affairs; **Mohammed Al Balwi**, King Saud bin Abdulaziz University for Health Sciences, Ministry of National Guard–Health Affairs, Riyadh; **Medhat Faris**, King Fahad Specialist Hospital, Dammam; **Ezzeldin Ibrahim**, Oncology Center of Excellence, International Medical Center, Jeddah, Kingdom of Saudi Arabia; and **Banu Arun**, The University of Texas MD Anderson Cancer Center, Houston, TX.

## Abstract

**Purpose:**

Over the past three decades, the incidence rate of breast cancer (BC) among Arab women has continually increased. However, data on the prevalence of *BRCA1/2* mutations are scarce. Although the population in Saudi Arabia is at large homogeneous and consanguinity is common, especially in the central, eastern, and southern regions of the country, the prevalence of *BRCA1* and *BRCA2* mutations and the characteristics of BC are not well studied in the country.

**Methods:**

This prospective observational study intended to determine the prevalence of *BRCA1* and *BRCA2* mutations and sought to examine the clinicopathologic features of BC associated with these mutations.

**Results:**

Of 310 patients, 270 (87%) had no mutation. *BRCA* mutations were identified in 40 patients; *BRCA1* mutations were found in 11% of patients, and *BRCA2* mutations were found in 2% of patients. Variants of unknown significance were found in 15% of patients (45 patients). Triple-negative BC (TNBC) accounted for 86% of all patients with BC and mutations. The following three recurrent deleterious founder *BRCA1* mutations were observed: c.4136_4137delCT was observed in five unrelated patients, c.5530delC was observed in three unrelated patients, and c.4524G>A mutations were observed in five unrelated patients. One novel mutation was identified in the *BRCA1* gene (c.5512 dup [p.Glu1838Glyfs*42]).

**Conclusion:**

Among high-risk Saudi patients with BC, *BRCA1* mutations are prevalent (11%). TNBC is the most common BC subtype. Furthermore, age alone does not have a significant association with mutation, but a combination of risk factors such as age, familial history, and TNBC has a significant association with *BRCA* mutation.

## INTRODUCTION

Breast cancer (BC) is the most common cancer in women across the world, with 1.7 million new BCs detected in 2012.^1,2^ Women in the Arab world are diagnosed with BC at more advanced stages, and the incidence rate has increased over the past three decades.^3-6^

In Saudi Arabia, BC ranks first among cancers in Saudi females, accounting for 27% of all newly diagnosed malignancies. Although the majority of BCs are sporadic, familial susceptibility to BC accounts for > 25% of all BCs.^7^

*BRCA1* and *BRCA2* gene mutations cause 20% to 25% of hereditary BCs^8^ and 5% to 10% of all BCs.^9^ Inheritance of germline mutations in *BRCA1* and *BRCA2* genes is autosomal dominant,^10^ and one in 400 to 1,000 persons in the general population are estimated to have a *BRCA1* or *BRCA2* mutation.^7^ In addition to BC, deleterious *BRCA1*/*2* mutations also predispose individuals to ovarian cancer; the average cumulative risk of ovarian cancer by age 70 years is 39% in *BRCA1* mutation carriers and 11% to 17% in *BRCA2* mutation carriers.^11,12^

Although some studies suggest that the prevalence of germline *BRCA1* and *BRCA2* mutations varies among ethnic groups and geographical areas,^7^ others conclude that mutation prevalence is similar across diverse races and ethnicities.^2,13^ However, some studies describe significant differences in the spectrum of *BRCA1* compared with *BRCA2* mutations and in *BRCA1/2* variants of uncertain significance.^2,13^ As such, women of African descent had the highest prevalence of variants of uncertain significance in a sample of 46,276 women of non–Ashkenazi Jewish ancestry (16.5% *v* 5.7% for Western European women; odds ratio, 3.2; 95% CI, 2.8 to 3.7),^13^ and *BRCA2* mutations were reported to be more frequent than *BRCA1* mutations in the Asian population.^14^ Population-specific mutations have also been described among Ashkenazi Jews^7^ as well as patients of Spanish ancestry.^15^ Founder *BRCA1* and *BRCA2* mutations have also been found in several European populations in Austria, Slovenia, Italy, France, Spain, Portugal, Belgium, the Netherlands (Holland), Germany, Czech Republic, Slovakia, Hungary, Greece, Cyprus, Denmark, Sweden, Norway, Finland, Iceland, the United Kingdom, Ireland, Poland, Latvia, Lithuania, Estonia, Belarus, and Russia.^16^

Although the population in Saudi Arabia is overall homogeneous and consanguinity is common, especially in the central, eastern, and southern regions of the country, the prevalence of *BRCA1* and *BRCA2* mutations and the characteristics of BC are not well studied. Available data are conflicting and inconclusive because they are based on retrospective analyses of small heterogeneous Saudi and non-Saudi patients.^17-22^Given these considerations, the main objectives of this study were to determine the prevalence and founder effect of *BRCA1* and *BRCA2* mutations in Saudi patients with BC and to study the clinicopathologic features of BC associated with these genetic mutations.

## METHODS

### Study Design

This prospective observational study enrolled patients between October 2010 and September 2016 at King Abdulaziz Medical City, Riyadh, Saudi Arabia. The study was approved by the Institutional Review Board of King Abdullah International Medical Research Center (RC12/158/R) and conducted in compliance with the International Conference on Harmonization Good Clinical Practice guideline.

### Eligibility and Enrollment

Patients with BC with at least one of the following high-risk criteria were eligible for the study: a first-degree relative with a known mutation in a cancer susceptibility gene; two or more BC primary tumors in a single family member; two or more individuals with breast cancer primary tumors on the same side of family with at least one family member diagnosed at age ≤ 50 years; ovarian cancer; male breast cancer; first- or second-degree relative with breast cancer at age ≤ 45 years; triple-negative BC (TNBC) at age < 60 years; and bilateral BC. After meeting the previously mentioned criteria, the patient or the substitute decision maker was approached for consent.

### Informed Consent

The study protocol and the informed consent were approved by the Institutional Review Board of King Abdullah International Medical Research Center, King Abdulaziz Medical City.

### Blood Collection, DNA Extraction, and Quantification

Approximately 3 mL of blood were collected in sterile tubes containing EDTA from all subjects enrolled onto the study. Genomic DNA was extracted following standard protocol and then screened for *BRCA1* and *BRCA2* mutations using next-generation sequencing. In addition, patients were screened for deletion or duplication genomic rearrangements within *BRCA* genes using multiple ligation probe amplification (MRC-Holland, Amsterdam, the Netherlands). Variants or mutations were validated by Sanger sequencing using specific polymerase chain reaction primers and sequenced on an ABI 3730 DNA Analyzer (Thermo Fisher Scientific, Waltham, MA). The blood samples were sent for testing at the Clinical Molecular and Personalized Diagnostics Unit at Catholic University and Hospital Foundations in Rome, Italy.

### Statistical Analysis

Categorical variables are reported as numbers and frequencies. χ^2^ and Fisher’s exact tests were performed as appropriate to assess any association between gene mutations and other parameters, with a two-sided significance level of 5%. The statistical analysis was carried out using IBM SPSS Statistics Version 22.0 (IBM, Armonk, NY).

## RESULTS

### Patient Characteristics

**Fig 1:**
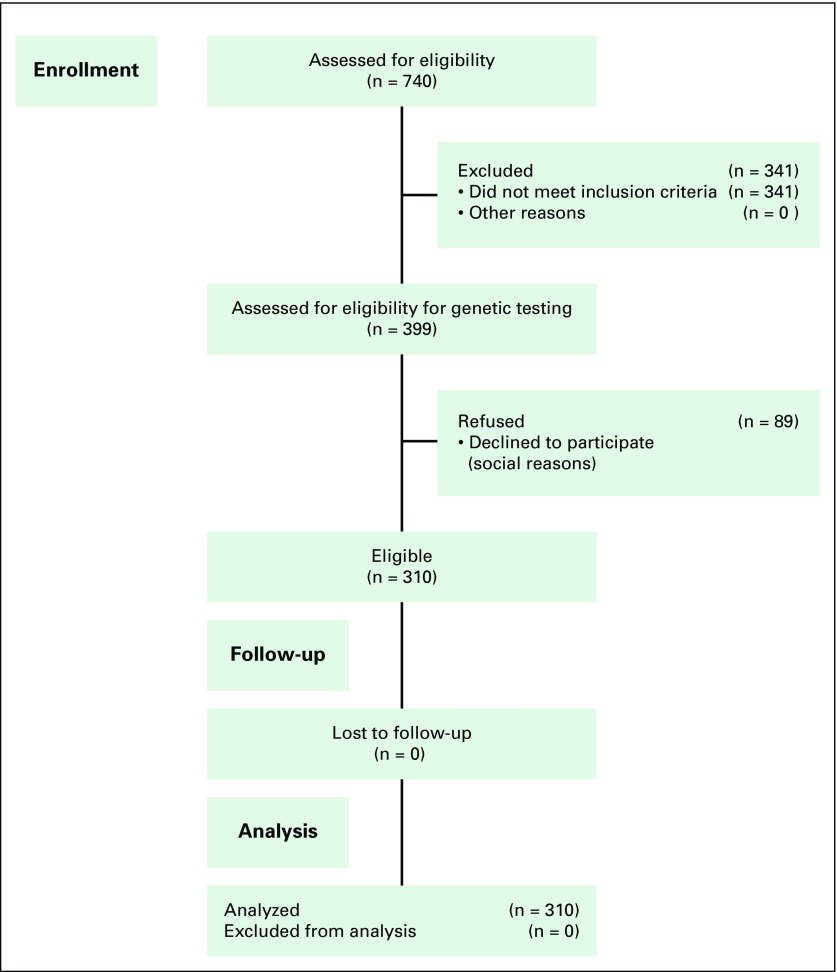
Flow diagram of enrolled patients.

Seven hundred forty Saudi patients were diagnosed with BC during the study period, of whom 399 (46%) were eligible for genetic testing. However, 89 patients declined genetic testing for various reasons. Thus, 310 patients were eligible for statistical analysis (Fig 1). Patient demographic characteristics are listed in Table 1.

**Table 1:**
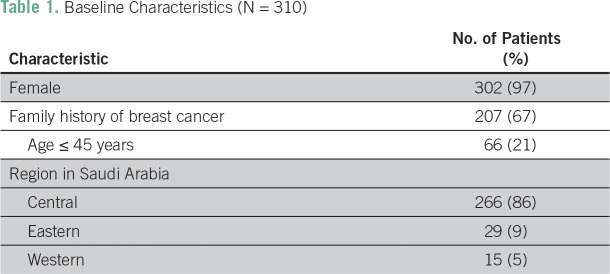
Baseline Characteristics (N = 310)

In total, 302 patients (97.4%) were women and eight (2.6%) were men. Sixty-six patients (21.3%) were younger than age 45 years. One hundred three patients (33.2%) had a family history of breast cancer.

### Disease Characteristics

Almost half of the patients (n = 153; 49.3%) had a stage II BC, and TNBC was the most common molecular subtype (n = 126; 40.6%). Nine patients (2.9%) had bilateral BC, and invasive ductal carcinoma was the most common histology (n = 289; 93.2%; Table 2).

**Table 2:**
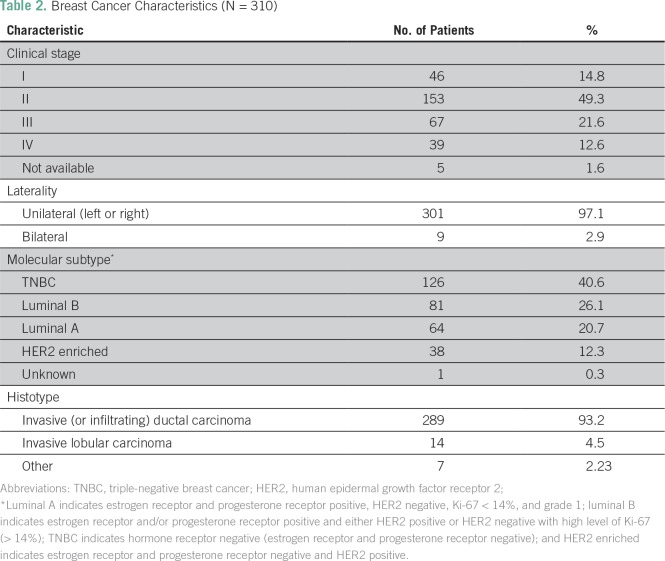
Breast Cancer Characteristics (N = 310)

### *BRCA* Mutations

Of 310 patients, 270 (87.1%) had no mutations. *BRCA1* or *BRCA2* mutations were identified in 40 patients (12.9%), whereas variants of unknown significance were reported in 45 patients (14.5%). *BRCA1* mutations (10.7%) were more prevalent than *BRCA2* mutations (2.2%; Table 3).

**Table 3:**
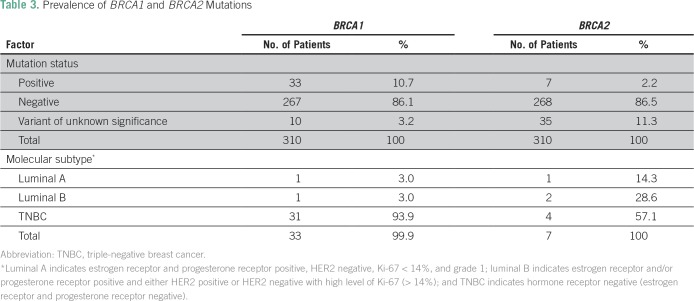
Prevalence of *BRCA1* and *BRCA2* Mutations

Four patients with BC and one patient with BC and ovarian cancer were reported to be carriers of the recurrent mutation c.4136_4137delCT (p.SER1379*). The five BCs had a triple-negative molecular profile and invasive ductal carcinoma histology; one patient had a family history of BC (first- and fourth-degree relatives). The c.4136_4137delCT (p.SER1379*) mutation accounted for 15% of *BRCA1* mutations, and the c.4524G>A (p.Trp1508Ter*) mutation was reported in another five patients (15%). In addition, we identified an unreported mutation (c.5512 dup [p.Val1838Glyfs*42]) in one family (mother and daughter with TNBC). The daughter was diagnosed first, at 28 years old, and the mother was diagnosed 2 years later (Table 4).

**Table 4:**
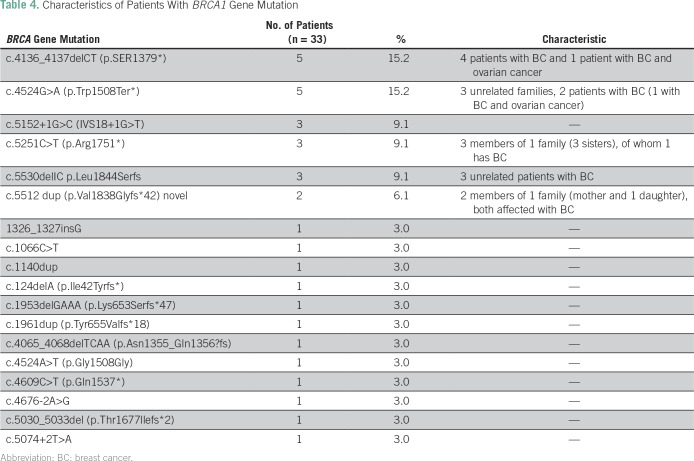
Characteristics of Patients With *BRCA1* Gene Mutation

Disease-associated *BRCA2* mutations were reported in seven patients, but none were identified more than once (Table 5). Forty-five patients (14.5%) had mutations of unknown significance; the majority were in *BRCA3* (Table 6).

**Table 5:**
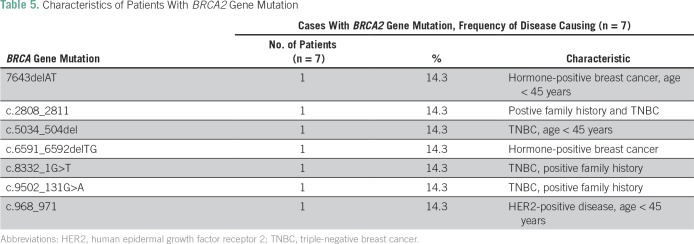
Characteristics of Patients With *BRCA2* Gene Mutation

**Table 6:**
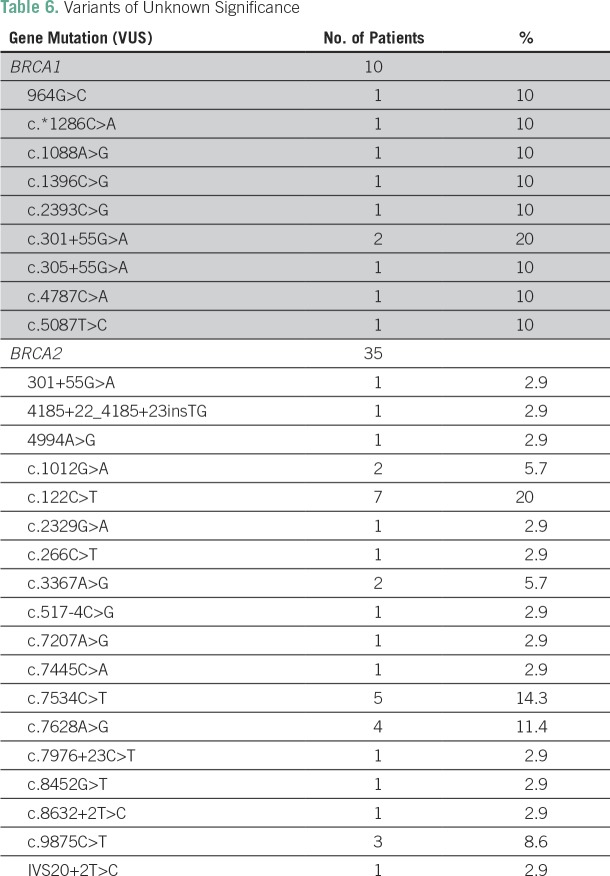
Variants of Unknown Significance

### Correlation of *BRCA1* Gene Mutation With Different Parameters

We determined the correlation between *BRCA1* gene mutations and different molecular subtypes and found that TNBC is highly associated with *BRCA1* mutations (*P* < .001) and family history of BC (*P* < .001) but that young age alone (≤ 45 years old, *P* = .358) was not associated with significant risk (Table 7). Overall, the *BRCA* mutation rate was significantly higher with two or more risk factors than with a single risk factor.

**Table 7:**
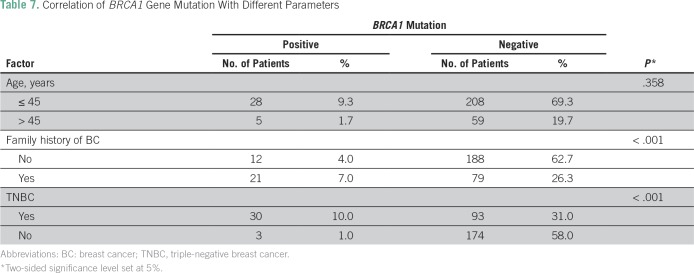
Correlation of *BRCA1* Gene Mutation With Different Parameters

## DISCUSSION

Little is known about the molecular analysis of *BRCA1* and *BRCA2* and etiologic factors of BC in Saudi Arabia.^14,21^ To our knowledge, the current study is the first prospective study in the country for *BRCA* testing in selected high-risk Saudi patients with BC. It showed that 12.9% of selected high-risk patients with BC had *BRCA* deleterious mutations, similar to frequencies reported from Lebanon^23^ but higher than frequencies reported from Qatar.^24^ In our study, *BRCA1* mutations were more common (82.5% of mutations) than *BRCA2* mutations, which is similar to the pattern in the Western population, although different from that among Asian populations, in which *BRCA2* mutations are more common.^2^

Three recurrent deleterious *BRCA1* mutations (c.4136_4137delCT, c.5530delC, and c.4524G>A) were identified. Likewise, a recent retrospective study published in 2016 reported three recurrent *BRCA1* mutations (c.1140dupG, c.4136_4137delCT, and c.5530delC) in 818 unselected patients with BC from different ethnicities in Saudi Arabia. These patients were diagnosed with primary BC between 1990 and 2011 and had their files and samples selected from another major medical and research center in the country (King Faisal Specialist Hospital and Research Centre, Riyadh, Saudi Arabia).

The current study identified an unreported mutation in *BRCA1* (c.5512dupG) in two patients with BC from one family, a 28-year-old daughter and her mother diagnosed 2 years apart, both with TNBC. This novel mutation, along with other reported founder mutations in Saudi Arabia, may contribute to the potentially disease-associated etiology of BC in Saudi Arabia. Such knowledge is also important for cost-effective genetic testing strategies for *BRCA1/2* gene mutations.

In our study, TNBC accounted for 85% of BCs in all patients with mutations (93.9% in patients with *BRCA1* mutations and 57.1% in patients with *BRCA2* mutations), which is consistent with the literature because TNBC is the predominant molecular profile in patients with a germline *BRCA1* mutations.^25,26^ Importantly, the literature reports similarity in gene expression profiles between TNBC and BC in women with *BRCA1* mutations.^27^ In addition, the current study shows that the frequency of *BRCA* mutation was significantly higher with two or more risk factors than one risk factor, which is comparable to the results of a local study conducted among patients with BC from different ethnicities and international studies.^28-30^ TNBC was the most important risk factor, followed by family history and young age. In addition, our results agree with data from international studies demonstrating that *BRCA* mutations are influenced by a TNBC molecular profile.^27,31,32^ Similar observations have been made with respect to young age, and the frequency of *BRCA* mutations seems to be increased in patients diagnosed at a young age.^28^

Finally, our study identified novel *BRCA* mutations and their predictors and risk factors. Such mutations seem to be specific for Saudi patients because different mutations are described in patients from Lebanon and North Africa.^23^ Our study also highlights challenges and limitations for *BRCA* testing in Saudi Arabia and possibly the whole Arab region. These include lack of a database for the Arab population, scarcity of local central laboratories and shortage of genetic counselors (only six genetic counselors are available in Saudi Arabia), social barriers to individuals’ acceptance, lack of awareness among patients and clinicians, and cost of testing. Therefore, future goals in Saudi Arabia are guided toward improving the strategy for genetic testing in BC. This strategy includes developing a cost-effective *BRCA* testing panel, creating a national and regional database, recruiting more genetic counselors, identifying high-risk patients for preventive services, and establishing an effective awareness and educational program. Additional efforts consist of implementing universal genetic screening guidelines in which a TNBC molecular profile and young age would be added as criteria. There was also a high rate of variants of unknown significance (87.5%) in our cohort, as well as other studies published from the region,^21,24,33^ and reporting these variants is important because some of them might be classified as pathogenic variants in the future.
